# Spreadable plant‐based cheese analogue with dry‐fractioned pea protein and inulin–olive oil emulsion‐filled gel

**DOI:** 10.1002/jsfa.11902

**Published:** 2022-04-13

**Authors:** Marina Mefleh, Antonella Pasqualone, Francesco Caponio, Davide De Angelis, Giuseppe Natrella, Carmine Summo, Michele Faccia

**Affiliations:** ^1^ Department of Soil, Plant and Food Science (DiSSPA) University of Bari Aldo Moro Bari Italy

**Keywords:** legume proteins, dry‐fractionation, emulsion‐filled gel, textural analysis, plant‐based cheese, volatile compounds

## Abstract

**BACKGROUND:**

Consumer demand for plant‐based cheese analogues (PCA) is growing because of the easy and versatile ways in which they can be used. However, the products available on the market are nutritionally poor. They are low in protein, high in saturated fat and sodium, and often characterized by a long list of ingredients.

**RESULTS:**

A clean label spreadable plant‐based cheese analogue was developed using dry‐fractionated pea protein and an emulsion‐filled gel composed of extra virgin olive oil and inulin, added in different concentrations as fat replacer (10%, 13% and 15% of the formulation). First, nutritional and textural analyses were performed, and the results were compared with two commercial products. The products were high in protein (134 g kg^−1^) and low in fat (52.2 g kg^−1^). The formulated PCAs had similar spreadability index to the dairy cheese but lower hardness (15.1 *vs.* 19.0 N) and a higher elasticity (0.60 *vs.* 0.35) consequent to their lower fat content (52.2 *vs.* 250 g kg^−1^). Then, dry oregano and rosemary (5 g kg^−1^) were added to the PCA, and sensory evaluation and analysis of volatile compounds were conducted. The addition of spices masked the legume flavor and significantly enriched the final product with aromatic compounds.

**CONCLUSION:**

The use of dry‐fractioned pea protein and of the emulsion‐filled gel allowed us to develop a clean label and nutritionally valuable spreadable plant‐based cheese analogue. Overall, the ingredients and product concepts developed could be used to upgrade the formulation of plant‐based cheese on a larger scale. © 2022 The Authors. *Journal of The Science of Food and Agriculture* published by John Wiley & Sons Ltd on behalf of Society of Chemical Industry.

## INTRODUCTION

Adopting a plant‐based diet is gaining considerable attention. It is supported by national health research organizations^1^ because it is considered more sustainable to the health and the environment than a regular diet including meat.

Today, the plant‐based food industry is facing a challenge in response to increased demand for innovative and nutritive plant‐based products.^2^, ^3^ Cheese represents an important food across the world and is produced in a wide diversity of flavors, textures and consumption patterns. Consequently, producing dairy‐free cheese analogue with a good nutritional profile mimicking the texture of a ‘real’ cheese is crucial for the dairy‐free food industry.^4^ For example, spreadable or soft cream cheese is a processed cheese adaptable to the fast‐food trade and widely consumed worldwide as a spread or as an ingredient in a variety of cold prepared foods. Soft creams usually have a high fat content (280 g kg^−1^).^5^ Therefore, the production of plant‐based spreadable cheese with better nutritional properties than its conventional counterpart is a challenge. Indeed, most plant‐based cheese analogues (PCA) on the market are nutritionally poor.^6^ They are low in protein content, usually having less than 50 g kg^−1^ protein.^4^, ^6^ Improvement of the protein content could be achieved by using pulse proteins. There is still a lack of knowledge regarding the ability of such proteins to mimic the technological properties of the casein matrix. Pulse proteins do not form a continuous protein network, and so the dairy‐free industry usually uses non‐protein ingredients, e.g. modified or non‐modified starches, gums and hydrocolloids, to fill the structural requirements and to stabilize the fat globules with starch.^4^, ^7^

Among the pulse proteins available on the market, dry‐fractionated peas concentrated in proteins are worthy of attention since they are extracted with no water and chemicals, and they are characterized by about 550 g kg^−1^ protein alongside carbohydrates, minerals and lipids.^8^ Such protein concentration has been proven to be suitable for the preparation of plant‐based meat analogues,^2^ demonstrating that the high protein content of commercial protein isolates (>800 g kg^−1^) is often unnecessary in food preparation. Moreover, the presence of carbohydrates, lipids and minerals can enhance the functionality as well as the nutritional composition of the final product. The drawback of dry‐fractionated pulse protein concentrates is the marked beany flavor, similar to the raw legumes, which is not always appreciated by consumers.^9^

In an attempt to mimic the properties of the fat present in cheese, most of the PCA found on the market contain unflavored coconut oil or palm oil.^6^, ^10^ As a result, PCA are high in saturated fat, which is associated with increased blood low‐density lipoprotein (LDL)‐cholesterol levels, often considered a marker for cardiovascular disease risk.^11^ In fact, Demmer *et al*.^12^ showed that the saturated fatty acids of a non‐dairy cheese alternative containing palm oil increased blood pro‐inflammatory markers even more than the saturated fatty acids of a dairy cheese. To overcome this issue, inulin could be used as a fat replacer, since it forms a white creamy and spreadable gel when mixed with water.^13^ Inulin has been used in many food categories including sausages,^14^, ^15^ dairy desserts,^16^ cheeses^17^ and biscuits.^18^

In this scenario, the aim of the present work was to formulate a spreadable, soft cream plant‐based cheese analogue, with improved nutritional composition compared with those already existing in the market. To achieve these goals, an emulsion‐filled gel (EFG) made with inulin and extra virgin olive oil (EVOO) and a dry‐fractionated pea protein with a high protein content were used. The nutritional and textural properties of the final products were analyzed and compared to two different commercial spreadable cheeses – a soy‐based cheese analogue and a conventional dairy cheese – available on the Italian market. Finally, to enhance the flavor of the final product, oregano and rosemary, two well‐appreciated spices in the Mediterranean region,^19^ were added and a sensory evaluation and volatile compound analysis were performed.

## MATERIAL AND METHODS

### Material

The dry‐fractionated pea protein (protein content of 560 g kg^−1^ dry matter (N × 6.25)) was obtained from Innovaprot SRL (Gravina in Puglia, Italy) and was produced from raw micronized flour of dehulled green pea (*Pisum sativum* L.).^8^ Tapioca starch was purchased from Molino Bongiovanni (Villanova Mondovì, Italy). Inulin was purchased from Farmalabor srl (Canosa di Puglia, Italy), xanthan (200 mesh) from Special Ingredients Europe (Garlenda, Italy) and the EVOO, dry oregano and dry rosemary from local producers in Apulia (Italy).

The non‐dairy cheese (NDC) from soy protein and the dairy cheese (DC) used for comparative analysis were purchased from a local supermarket in Bari, Italy. NDC was composed, as per the list of ingredients reported on the packaging, of fermented soy protein (water 750 mL kg^−1^ and soybeans 84 g kg^−1^) live cultures, coconut oil, dietary fiber, carrageenan–sodium alginate, pectin, calcium phosphate, sea salt and vitamin D2; while DC was composed of pasteurized milk, cream, salt, sodium alginate, flour of bean seeds and carrageenan. The nutritional composition of NDC, according to the nutritional facts mentioned on the package, was as follows (in 100 g); energy 211 kcal, fat 20 g, carbohydrates 4.5 g, sugar 1.3 g, protein 2.9 g, salt 0.89 g and calcium 120 mg; while that of DC was as follows (in 100 g); energy 249 kcal, fat 25 g, carbohydrates 2.9 g, sugar 2.9 g, protein 3.7 g and salt 0.74 g.

### Pea‐based cheese formulations

#### Preparation of EFG

**Figure 1:**
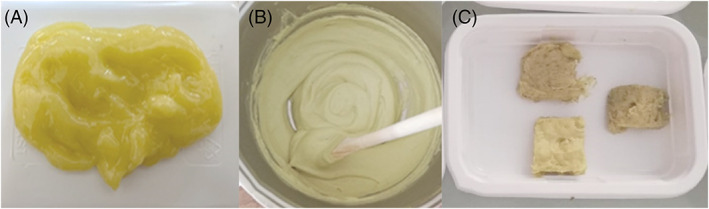
(A) Emulsion‐filled gel produced from EVOO, inulin, water and xanthan gum. (B) Slurry made with the dry‐fractionated pea protein and water (30:70, w/v). (C) Plant‐based cheese analogues PCA‐HG (bottom), PCA‐HGO with oregano (top left) and PCA‐HGR with rosemary (top right).

The EFG (Fig. 1) was produced from EVOO, inulin, water and xanthan, according to a revised procedure reported in Paradiso *et al*. (2015),^13^ and was used in this study as a replacement to high‐saturated fats such as coconut or palm oil. The composition of the EFG was as follows: EVOO 370 mL kg^−1^, inulin 190 g kg^−1^, water 420 mL g kg^−1^ and xanthan 20 g kg^−1^.

### Preparation of the slurry

Dry‐fractionated pea protein was slowly added to the water while mixing and heating to eliminate eventual lumps. The slurry (Fig. 1) was composed of 300 g kg^−1^ dry‐fractionated pea protein and 700 mL kg^−1^ water, and was heated for 40 min at 80 °C. The slurry had a pH of 5.9 and a moisture content of 680 g kg^−1^, and contained 15 g kg^−1^ ash, 7.3 g kg^−1^ fat, 110 g kg^−1^ carbohydrates, 8 g kg^−1^ sugar and 180 g kg^−1^ protein.

### Preparation of pea‐based cheese analogues

Three formulations of PCAs were prepared with different slurry and EFG percentages. Once the slurry was cooled, the EFG was added in different concentrations: low gel (PCA‐LG) contained 820 g kg^−1^ slurry and 100 g kg^−1^ EFG; medium gel (PCA‐MG) contained 790 g kg^−1^ slurry and 130 g kg^−1^ EFG; and high gel (PCA‐HG) contained 770 g kg^−1^ slurry and 150 g kg^−1^ EFG. In addition, the three formulations contained 70 g kg^−1^ tapioca^20^ and 10 g kg^−1^ salt. The EFG percentage ranges were chosen considering in preliminary trials the spreadability index of the final products, evaluated by an internal panel. When the EFG percentage was less than 100 g kg^−1^ the product was too liquid, and when it was higher than 150 g kg^−1^ the product had a greasy texture. The ingredients were mixed together and heated for 20 min at 40 °C. After cooling, the mixture was poured into rectangular molds and stored at 4 °C.

### Chemical analyses

Moisture content was determined using a thermal balance (MAC 110/NP, Radwag, Radom, Poland) and pH was evaluated by means of a pH meter (Edge HI2002, Hanna Instruments, Columbus, OH, USA). The lipid content was determined by the Soxhlet method following the AACC method.^21^ The ash content was determined as per the AACC method.^22^ Protein content (N × 6.25) was determined by the Kjeldhal method.^23^ Total energy in kilocalories was calculated using the Atwater conversion factors.^24^ For determination of sugars – fructose, galactose, glucose, lactose, maltose and saccharose – samples were analyzed by high‐performance liquid chromatography coupled with refractive index detection on an Agilent apparatus (Agilent, Santa Clara, CA, USA) equipped with a Spherisorb Amino (NH2) column 80 Å, 5 mm, 4.6 mm × 250 mm (Waters, Milford, MA, USA). The separation was done under isocratic conditions as reported by Trani *et al*.^25^ using acetonitrile–water (70:30, v/v) as mobile phase at a constant flow rate (1.8 mL min^−1^). Total sugar content has been expressed as the sum of the single sugars analyzed. For the analyses of total calcium (Ca), sodium (Na) and potassium (K) content, samples were digested using a Multiwave 3000 (Anton Parr, Graz, Austria) based on UNI EN 13805:2014^26^ standards and analyzed using a NexION ICP mass spectrometer (PerkinElmer, Waltham, MA, USA). Samples were mineralized with nitric acid (Suprapur, Merck, Kenilworth, NJ, USA) at an amount of 10 mL 0.5 g^−1^ sample. The process was carried out in Teflon containers at a temperature of 200 °C for 40 min by means of a high‐pressure microwave method (MarsXPres, CEM, Stallings, NC, USA). Potassium and sodium were determined by adding buffer solution of Schuhknecht and Schinkel (cesium chlorate and aluminum nitrate at concentrations of 50 and 250 g L^−1^, respectively), while calcium content was determined by adding a buffer solution of Schinkel (cesium chloride and lanthanum chloride at a concentration of 10 g L^−1^). Minerals were determined at the following wavelengths: 766.5 nm for K, 222.7 nm for Ca and 589.0 for Na. Mineral content is given in g kg^−1^ of the final product.

### Color and texture profile analysis

Lightness (L*), green–red (a*) and blue–yellow (b*) were measured using a model CM‐600d Konica Minolta colorimeter (Osaka, Japan). The spreadability index of the cheese was determined directly after removing it from the cell at 4 °C, as described by Anitha and Satyanarayana.^27^ Briefly, 0.5 g of the PCA was placed within a circle of 1 cm diameter pre‐marked on a glass plate, over which a second glass plate was placed. A weight of 500 g was allowed to rest on the upper glass plate for 5 min. The increase in diameter due to spreading of the cream was noted.

Texture profile analysis (TPA) was determined on samples of 2 cm thick slices (3.5 cm × 3.5 cm) directly after removal from the cell at 4 °C, using a Z1.0 TN texture analyzer (Zwick Roell, Ulm, Germany) with a 5 kg load cell, equipped with a 36 mm diameter cylindrical probe. TPA conditions in the cyclic compression test were: 1 mm s^−1^ compression; 50% sample deformation in both compressions; and 5 s pause before the second compression. The reported values represent the averages of six replicates. Data were acquired by means of TestXPertII version 3.41 software (Zwick Roell). Hardness was reported as peak maximum force (in N) upon first compression. The other parameters reported – springiness, gumminess, chewiness and cohesiveness – were defined as reported in Summo *et al*.^28^

### Preparation of spiced PCAs

For sensory analysis, PCA with 150 g kg^−1^ EFG (PCA‐HG) was selected, after it was shown to have a similar spreadability index to the commercial DC, and spiced with oregano or rosemary. The method of preparation was as follows: after mixing the slurry well with 150 g kg^−1^ EFG, 5 g kg^−1^ dry oregano (PCA‐HGO) or 5 g kg^−1^ dry rosemary (PCA‐HGR), the mixture was molded and refrigerated at 4 °C. The percentage of oregano and rosemary was chosen by trained sensory panelists after evaluating three different concentrations (5, 7.5 and 10 g kg^−1^).

### Analysis of volatile organic compounds

Volatile organic compounds (VOC) were extracted by the solid‐phase microextraction (SPME) technique. Analytes were separated and identified from pea slurry, PCA‐HG, PCA‐HGO and PCA‐HGR by gas chromatography–mass spectrometry (TRACE 1300 gas chromatograph, ISQ Series 3.2 SP1 single‐quadrupole mass spectrometer, Thermo Scientific, Rodano, Italy) equipped with a VF‐WAX MS column (60 m × 0.25 mm, 0.25 μm). Vials containing 1 ± 0.05 g of each sample were loaded into an autosampler (Triplus RSH, ThermoFisher Scientific, Rodano, Italy). Each of them was incubated at 50 °C for 40 min; then a divinylbenzene/carboxen/polydimethylsiloxane (DVB/CAR/PDMS) 50/30 mm SPME fiber assembly (Supelco, Bellefonte, PA, USA) was inserted into the vial headspace for the extraction phase (60 min). The fiber was then desorbed at 220 °C × 2 min with ultra‐high‐purity helium (carrier gas operating at 1 mL min^−1^ flow rate) into the injection port in a splitless mode. The analysis conditions were as follows: initial oven temperature of 40 °C (maintained for 5 min) was ramped at 4 °C min^−1^ to 140 °C; then at 10 °C min^−1^ increased rate to 210 °C and held at that temperature for 7.5 min for a total run time of 45 min. The mass detector was set as follows: detector voltage, 1700 V; source temperature, 250 °C; ionization energy, 70 eV; scan range, 33–200 amu. Volatile compounds were identified by comparison between analyte spectra and reference mass spectra of the NIST library (NIST 2.0 mass spectrometry database). Results were expressed as percentage of area of each peak from the total peak area. Values are mean area units (AU) (divided by 10^6^) of two replicates from each product.

### Quantitative descriptive analysis of sensory characteristics

Quantitative descriptive analysis of sensory features involved the presentation of the three plant‐based cheese samples, a non‐spiced plant‐based cheese analogue (PCA), a plant‐based cheese analogue with oregano (PCA‐HGO) and a plant‐based cheese analogue with rosemary (PCA‐HGR) (Fig. 1), which were randomly coded to a panel of 13 members who were familiar with spreadable cheese as described by Pasqualone *et al*.^29^ The sensory panelists (seven males, six females; age range 25–52) were recruited based on their previous experience in sensory evaluation among professors, technicians and researchers at the laboratory of the Food Science and Technology unit of the Department of Plant, Soil and Food Sciences of the University of Bari, Italy. Pre‐test sessions were carried out to define the list of descriptors to be evaluated and the intensity range, and to verify reliability, consistency and discriminating ability of panelists when testing the products. The study protocol followed the ethical guidelines of the laboratory. Panelists were given information about study aims and individually written informed consent was obtained from each participant. All tested samples were of food grade. Panelists rated the products, giving a score of 0 (absence of attribute), 1 (poor sensation), 2 (mild sensation) or 3 (strong sensation) for parameters of odor, describing the orthonasal and retronasal sensation felt, including grass, plant, inulin, legumes, spices, oregano and rosemary; taste: sweet, salt, bitter, sour or astringent; and texture: lumpy, creamy, sticky, gummy or soluble.

### Statistical analysis

All data were subjected to one‐way analysis of variance, followed by Tukey's HSD (honestly significant difference) test at *α* = 0.05 using R software.^30^ To compare the PCA formulations to commercial NDC or DC, Dunnett's multiple comparisons test was carried out at *α* = 0.05. The formulations were replicated three times by producing three different slurries. Each sample was analyzed twice, except for TPA parameters, which were repeated six times. Data were expressed as means ± standard deviation.

## RESULTS

### Chemical analysis and nutritional composition of the three formulations PCA‐HG, PCA‐MG and PCA‐LG

Moisture and ash contents and pH were not different among the three formulations. Moisture ranged between 592 and 624 g kg^−1^ (data not reported), while the average pH and ash content values were 5.6 and 23 g kg^−1^, respectively (Table 1).

**Table 1:** Fat, protein, carbohydrate and sugar content expressed as g kg^−1^ and the total energy, expressed in kilocalories (kcal), sodium (Na), potassium (P) and calcium (Ca) content and the Na:K ratio of the three formulations with high (PCA‐HG), medium (PCA‐MG) and low (PCA‐LG) emulsion‐filled gel concentration. Data are expressed as means ± SD

	PCA‐HG	PCA‐MG	PCA‐LG
Fat (g kg^−1^)	60.3 ± 4.5a	53.3 ± 0.7ab	43.0 ± 1.7b
Proteins (g kg^−1^)	130 ± 3	133 ± 9	140 ± 4
Carbohydrates (g kg^−1^)	195 ± 4a	180 ± 9ab	170 ± 8b
Sugar (g kg^−1^)	7.00 ± 0.70	6.90 ± 0.10	6.10 ± 0.00
Ash (g kg^−1^)	23.0 ± 0.2	23.0 ± 0.2	23.0 ± 0.1
Calories (kcal)	736 ± 6a	692 ± 7ab	652 ± 3b
pH	5.60 ± 0.00	5.60 ± 0.00	5.60 ± 0.00
Na (g kg^−1^)	4440 ± 23	4240 ± 26	4090 ± 26
K (g kg^−1^)	4830 ± 31	4920 ± 33	5170 ± 40
Ca (g kg^−1^)	590 ± 6.4	600 ± 6.2	600 ± 5.7
Na:K	0.92 ± 0.05a	0.86 ± 0.02ab	0.79 ± 0.03b

Means with the same letter are not statistically different with Tukey's test at *P* ≤ 0.05.

The content of fat, carbohydrate and protein are reported in Table 1. As expected, PCA‐HG had the highest fat content (60.3 g kg^−1^), followed by PCA‐MG (53.3 g kg^−1^) and PCA‐LG (43.3 g kg^−1^). Carbohydrate content was significantly higher in PCA‐HG than PCA‐LG, with no differences in PCA‐MG. Protein content was not affected by different concentrations of EFG. Sugar content of PCA‐HG, PCA‐MG and PCA‐LG ranged from 6.1 and 7.0 g kg^−1^, without significant differences among the PCAs.

PCAs had different total energy values, with PCA‐HG having the highest value (736 kcal), followed by PCA‐MG (692 kcal) and PCA‐LG (652 kcal) (Table 1).

The macro elements analyzed were sodium (Na), potassium (K) and calcium (Ca). Evidently, their contents were not significantly different among the three different formulations. The formulated PCAs had a mean content of 4260 g kg^−1^ Na, 595 g kg^−1^ Ca and an Na:K ratio ranging from 0.79 to 0.92 (Table 1).

### Texture analysis and lightness of the three formulations PCA‐HG, PCA‐MG and PCA‐LG and of commercial DC and NDC

The textural properties of the formulations are reported in Table 2. The spreadability index, hardness and springiness were significantly affected by the addition of rising concentrations of EFG. In particular, PCA‐HG had higher spreadability than PCA‐LG and the same value (0.60 cm) of DC. NDC had the highest spreadability index of all the analyzed samples (1.37 cm).

**Table 2:** Spreadability index, hardness, springiness, gumminess, chewiness, cohesiveness and lightness index (*L*) of the three formulations, high emulsified gel filler (PCA‐HG), medium emulsified gel filler (PCA‐MG) and low emulsified gel filler (PCA‐LG) and of the commercial dairy cheese (DC) and non‐dairy cheese (NDC). Data are expressed as means ± SD

	Spreadability index (cm)	Hardness (N)	Springiness	Gumminess (N)	Chewiness (N)	Cohesiveness	L*	a*	b*
PCA‐HG	0.60 ± 0.04a	13.2 ± 1.0b	0.73 ± 0.01a	3.78 ± 0.55	3.05 ± 0.42	0.58 ± 0.09	61.5 ± 1.1	0.55 ± 0.08	27.0 ± 0.8
PCA‐MG	0.54 ± 0.04ab	16.4 ± 1.1a	0.59 ± 0.04b	4.56 ± 0.50	2.98 ± 0.61	0.45 ± 0.07	61.5 ± 1.4	0.51 ± 0.08	26.9 ± 0.3
PCA‐LG	0.49 ± 0.04b	15.8 ± 1.1a	0.53 ± 0.05b	4.57 ± 0.76	2.83 ± 0.45	0.45 ± 0.04	61.2 ± 0.8	0.58 ± 0.07	27.0 ± 0.9
DC	0.60 ± 0.05a	19.2 ± 0.3^	0.35 ± 0.05^	0.81 ± 0.05^	0.29 ± 0.08^	0.25 ± 0.03^	86.6 ± 0.3^	0.15 ± 0.05^	10.39 ± 0.7^
NDC	1.73 ± 0.10^	17.9 ± 0.2a	0.90 ± 0.01^	0.34 ± 0.03^	0.32 ± 0.07^	0.24 ± 0.01^	78.6 ± 0.2^	0.94 ± 0.08^	12.35 ± 0.6^

Means with the same letter are not statistically different with Tukey's test at *P* ≤ 0.05.

^Significantly different from the values of the three formulations of PCA and their average with Dunnett's test at *P* ≤ 0.05.

DC had the highest hardness value (19.1 N) of all the samples studied. Among the formulated PCAs, PCA‐HG (13.24 N) had a lower hardness than PCA‐MG and PCA‐LG (16.4 and 15.8 N respectively) and a similar hardness to NDC (17.9 N).

DC had the lowest springiness value (0.35) of all the samples studied, while NDC had the highest one (0.90). The springiness of PCA‐MG and PCA‐LG were significantly lower (0.53 and 0.59) than that of PCA‐HG (0.73).

No significant differences were found among PCAs for gumminess (3.78–4.57), chewiness (2.83–3.05 N) and cohesiveness (0.45–0.58). However, all the PCAs produced had significantly higher gumminess, chewiness and cohesiveness than DC (0.81, 0.29 and 0.25, respectively) and NDC (0.34, 0.32 and 0.24, respectively).

The three color indices – green–red (a*), blue–yellow (b*) and lightness (L*) – were not different among the three PCAs but significantly different from NDC and DC (Table 2). The formulated products had a light‐green color, while NDC had a beige color and DC a white color. As seen in Table 2, the three PCAs had a significantly lower L* index than NDC and DC, and higher b* index than NDC and DC.

### VOC of slurry, PCA‐HG, PCA‐HGO and PCA‐HGR

**Figure 2:**
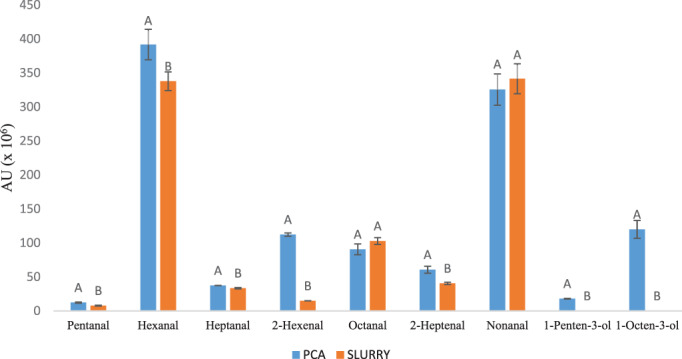
Volatile aroma compounds derived from the lipoxygenase activity found in the slurry and plant‐based cheese analogue high gel (PCA‐HG) without spices. Different letters indicate significant differences at *P* ≤ 0.05. Data are expressed as mean ± SD.

Figure 2 reports the principal component analysis of the VOC profiles of plant‐based cheese analogue high‐gel (PCA‐HG) and slurry. The addition of EFG to the slurry caused a significant increase in VOC amounts, which can be related to the presence of EVOO – mainly pentanal, hexanal, heptanal, 2‐hexenal, 2‐heptenal, 1‐pent‐3‐ol and 1‐octen‐3‐ol.

In Table 3, the main volatile compounds detected in PCA‐HG, PCA‐HGO and PCA‐HGR are reported. The volatile profiles of the three samples were significantly different. Many VOC were present only in PCA‐HGO, such as durenol, terpinene, thymol methyl ether, carvone, carvenone, terpinen‐4‐ol, α‐ylangene, *p*‐cymen‐7‐ol and 1‐heptanol. Moreover, *O*‐cymene and carvacrol were higher in PCA‐HGO (11‐ and 59‐fold, respectively) than in PCA‐HGR and absent in the plain PCA‐HG. The most abundant VOC found in PCA‐HGR were camphor, verbenone, eucalyptol, borneol, α‐linalool, thymol, camphene, *trans*‐carveol, 1‐penten‐3‐ol ethyl vinyl carbinol, α‐terpineol, pinocarvone, d‐limonene, 3‐carene and α‐pinene. PCA‐HG was the sample with the poorest number of VOC. It had the highest amounts of decanal, ethanol, 1‐penten‐3‐ol, dimethyl‐disulfide and γ‐caprolactone. Regarding the VOC total abundance, PCA‐HGO had the highest area unit (AU), followed by PCA‐HGR, PCA‐HG and slurry.

**Table 3:** The most representative volatile compounds found in plant‐based cheese analogue (PCA), plant‐based cheese analogue high gel with oregano (PCA‐HGO) and plant‐based cheese analogue high gel with rosemary (PCA‐HGR), expressed as area unit (AU) × 10^6^ Data are expressed as means ± SD

Compound	PCA	PCA‐HGO	PCA‐HGR
2.3‐Dimethylpentanal	0.00 ± 0.0b	0.00 ± 0.0b	4.50 ± 0.0a
Pentanal	12.29 ± 0.8b	12.27 ± 1.7b	16.24 ± 0.4a
Hexanal	392 ± 22.4b	293 ± 3.3c	455 ± 21.2a
2‐Pentenal	4.81 ± 0.2c	6.15 ± 0.2b	9.47 ± 0.2a
Heptanal	37.42 ± 0.2a	21.92 ± 1.9b	41.19 ± 4.2a
*trans*‐2‐Hexenal	112 ± 2.4b	201 ± 21.3a	242 ± 25.1a
Octanal	90.55 ± 6.3b	75.78 ± 7.9b	105 ± 5.9a
2‐Heptenal	60.63 ± 5.2b	58.63 ± 6.1b	74.63 ± 3.5a
Nonanal	3254 ± 36.4a	371.1 ± 11.3a	443.1 ± 103.1a
2‐Octenal	0.00 ± 0.0b	25.68 ± 1.8a	0.00 ± 0.0b
Decanal	21.58 ± 4.1a	0.00 ± 0.0b	0.00 ± 0.0b
2‐Nonenal	9.26 ± 0.7b	58.61 ± 3.3a	0.00 ± 0.0c
Ethanol	4.73 ± 0.1a	0.00 ± 0.0c	2.55 ± 0.6b
1‐Penten‐3‐ol	18.00 ± 0.5a	0.00 ± 0.0b	0.00 ± 0.0b
1‐Hexanol	241 ± 9.6ab	205 ± 26.4b	249 ± 1.2a
3‐Hexen‐1‐ol	56.61 ± 2.9c	77.80 ± 2.6a	72.36 ± 0.2b
1‐Octen‐3‐ol	26.07 ± 4.3b	0.00 ± 0.0c	225.49 ± 5.8a
1‐Heptanol	0.00 ± 0.0b	39.75 ± 3.6a	0.00 ± 0.0b
2‐Ethyl‐1‐hexanol	0.00 ± 0.0b	0.00 ± 0.0b	14.93 ± 2.7a
*p*‐Cymen‐7‐ol	0.00 ± 0.0b	5.14 ± 5.1a	0.00 ± 0.0b
Benzyl alcohol	0.00 ± 0.0c	31.13 ± 0.4b	37.12 ± 1.5a
Ethyl acetate	1.16 ± 0.1b	0.00 ± 0.0c	1.82 ± 0.1a
α‐Pinene	0.00 ± 0.0b	0.00 ± 0.0b	80.56 ± 6.5a
α‐Phellandrene	0.00 ± 0.0c	31.11 ± 6.7a	6.70 ± 0.5b
3‐Carene	0.00 ± 0.0c	17.24 ± 1.2b	53.96 ± 3.4a
d‐Limonene	22.05 ± 6.5c	155 ± 18.9b	330 ± 49.1a
Terpinene	0.00 ± 0.0b	1418 ± 431.3a	0.00 ± 0.0b
*o*‐Cymene	0.00 ± 0.0c	3191 ± 640.4a	272.6 ± 13.8b
α‐Ylangene	0.00 ± 0.0b	33.19 ± 0.6a	0.00 ± 0.0b
α‐Linalool	0.00 ± 0.0b	0.00 ± 0.0b	505.40 ± 7.1a
Pinocarvone	0.00 ± 0.0b	0.00 ± 0.0b	24.64 ± 1.0a
Terpinen‐4‐ol	0.00 ± 0.0b	3284 ± 0.7a	0.00 ± 0.0b
α‐Terpineol	0.00 ± 0.0b	0.00 ± 0.0b	155.51 ± 0.2a
Borneol	0.00 ± 0.0c	134.6 ± 15.5b	587.4 ± 12.0a
1‐Penten‐3‐ol ethyl vinyl carbinol	0.00 ± 0.0b	0.00 ± 0.0b	2.80 ± 0.1a
Verbenone	0.00 ± 0.0b	0.00 ± 0.0b	2032 ± 9.6a
Carvenone	0.00 ± 0.0b	67.90 ± 4.8a	0.00 ± 0.0b
Carvone	0.00 ± 0.0b	54.66 ± 0.7a	0.00 ± 0.0b
*t*‐Carveol	0.00 ± 0.0b	0.00 ± 0.0b	3.43 ± 0.2a
Eucalyptol	7.21 ± 0.5b	0.00 ± 0.0c	98 768 ± 58.6a
Camphene	6.12 ± 0.2c	14.13 ± 1.2b	29.57 ± 1.7a
Maltol	12.19 ± 3.0a	0.00 ± 0.0b	17.64 ± 2.8a
Thymol	0.00 ± 0.0c	7.36 ± 3.4b	52.15 ± 5.3a
Durenol	0.00 ± 0.0b	3455 ± 40a	0.00 ± 0.0b
Carvacrol	0.00 ± 0.0c	1855 ± 250a	31.35 ± 4.1b
Camphor	0.00 ± 0.0b	0.00 ± 0.0b	1667 ± 62.4a
6‐Methyl‐5‐hepten‐2‐one	31.97 ± 2.5ab	27.24 ± 2.2b	35.96 ± 3.2a
Acetic acid	39.66 ± 0.0b	45.04 ± 9.6ab	58.01 ± 6.6a
Dimethyl disulfide	2.53 ± 0.a	1.85 ± 1.6ab	1.35 ± 0.2b
γ‐Caprolactone	10.24 ± 0.1a	0.00 ± 0.0b	0.00 ± 0.0b
Thymol methyl ether	0.00 ± 0.0b	62.28 ± 2.5a	0.00 ± 0.0b
Total	2055 ± 145.7c	13 033 ± 1054.6a	9416 ± 153.2b

Means with the same letter are not statistically different with Tukey's test at *P* ≤ 0.05.

### Sensory evaluation of PCA‐HG, PCA‐HGO and PCA‐HGR

The results of the sensory evaluation of the three samples PCA, PCA‐HGO and PCA‐HGR are reported in Table 4. Regarding the odor attributes, PCA‐HG had higher scores for inulin (1.23) and legumes (1.69) than PCA‐HGO (0.44 and 0.77) and PCA‐HGR (0.40 and 0.92). The plant and grass mean scores were not different among the three samples.

**Table 4:** Sensory evaluation scores for plant‐based cheese analogue high‐gel (PCA‐HG), plant‐based cheese analogue high gel with oregano (PCA‐HGO) and plant‐based cheese analogue high gel with rosemary (PCA‐HGR)

	Parameter	PCA‐HG	PCA‐HGO	PCA‐HGR
*Odor*	Grass	1.38	0.92	1.15
	Plant	1.31	0.85	0.85
	Inulin	1.23a	0.40b	0.40b
	Legumes	1.69a	0.77b	0.92b
	Spices	0.00b	2.08a	2.00a
	Oregano	0.00b	2.39a	0.00b
	Rosemary	0.00b	0.23b	2.38a
*Taste*	Sweet	1.38	1.38	1.62
	Salt	1.08	0.92	1.15
	Bitter	0.00b	0.77a	0.00b
	Sour	0.00	0.00	0.00
	Astringent	0.00	0.00	0.00
	Lumpy	0.00b	0.85a	1.00a
*Texture*	Creamy	1.08	1.23	1.46
	Sticky	1.38	1.15	0.85
	Gummy	1.08	1.23	0.62
	Soluble	0.77	0.77	0.85

Means with the same letter are not statistically different with Tukey's test at *P* ≤ 0.05.

The four scores represent: 0, absence of attribute; 1, poor sensation; 2, mild sensation; 3, strong sensation.

Spices were mildly present in the aromatized products PCA‐HGO (2.08) and PCA‐HGR (2.00). Oregano and rosemary were added separately to the list of attributes to test whether panelists could feel and guess the right added spice. PCA‐HGO scored 2.39 for oregano and 0 for rosemary, whereas PCA‐HGR scored 2.38 for rosemary and 0.23 for oregano.

Sour and astringent tastes were not detected in the three samples. Bitterness was only detected in the PCA‐HGO, with a score of 0.77. The three samples had similar mean scores for sweet, ranging from 1.38 to 1.62, and for salt, ranging from 0.92 to 1.15. PCA‐HG, PCA‐HGO and PCA‐HGR were comparable for all texture scores except for lumpiness, which was detected in PCA‐HGO and PCA‐HGR.

## DISCUSSION

### Nutritional characteristics of PCA‐LG, PCA‐MG and PCA‐HG

The formulated plant‐based cheese analogues with different concentrations of EFG displayed a good nutritional composition in terms of protein, fat and total calories when compared to bovine DC and common plant‐based cheese analogues from soy and nuts.^3^

The formulated PCAs provided less energy than the commercial products, with DC being more calorific (996 kcal) than NDC (844 kcal). PCAs had a higher content of protein – more than 3 times – than DC, NDC and other commercial cream or spreadable cheeses which, according to the United States Department of Agriculture (USDA), have an average protein content of 70 g kg^−1^.^5^ The protein content of the formulated PCAs contributed 28% to 34% of the total calories, while the protein contents of DC and NDC were drastically lower (37 and 29 g kg^−1^, respectively) than the formulated ones and contributed to 5% of the total calories. As a result, the nutritional claim ‘high in protein’ can be granted to the formulated PCAs, as more than 20% of the total calories were from proteins.^31^ The addition of these claims is of importance, as they affect positively consumers' behavior towards purchase.^32^ This supports the current research on dry‐fractionated protein, confirming that this ingredient can be used for dairy alternatives, highlighting its potential in the production of plant‐based food. This outcome should encourage both the academic and industrial sectors to focus more on complex ingredients such as dry‐fractionated legume protein, which are more sustainable and less resource demanding than the protein isolates available on the market.^2^, ^8^ The carbohydrate content of PCAs was higher than those of DC (31.0 g kg^−1^) and NDC (53.0 g kg^−1^); however, the sugar content was lower than those of DC (29.0 g kg^−1^) and NDC (13.0 g kg^−1^), meaning that the higher carbohydrate content of the formulated PCAs is derived from complex carbohydrates, polysaccharides and oligosaccharides.

The addition of EFG to the formulation of the cheese analogue helped in producing a product with a lower fat content when compared to the cheese analogues already found on the market. Indeed, the fat percentages of the three formulated products were much lower than those of DC (250 g kg^−1^) and NDC (200 g kg^−1^). In fact, the formulated PCAs had less than 30% of their calories from fat, while NDC and DC had 90% and 85%, respectively, of their calories from fat. In the case of DC, the fat is from cow's milk and there is a high ratio of saturated over unsaturated fat. In the case of NDC, the fat is mainly from the saturated fat of coconut oil. According to the USDA, commercial cream or spreadable cheese, in general, has 280 g kg^−1^ fat, with 180 g kg^−1^ as saturated fat (SFA), 90 g kg^−1^ monounsaturated fat (MUFA) and 10 g kg^−1^ polyunsaturated fat (PUFA).^5^ The fat content of the formulated PCAs is derived mainly from olive oil, which has an elevated amount (up to 850 g kg^−1^) of unsaturated fatty acid in its lipidic fraction,^33^ thus respecting the recent draft guidelines published by the World Health Organization (WHO)^34^ recommending a reduction in SFA intake and its replacement with PUFA and MUFA.

Na:K intake ratio might be a better predictor of hypertension and cardiovascular events than sodium or potassium intake per se.^35^ Our PCAs satisfied the international recommendations set by the WHO and National Institutes of Health (NIH) for Na:K ratio: a molar ratio of less than 1.^36^, ^37^, ^38^ A study on the trend of potassium intake and Na:K ratio showed a high intake of Na and an insufficient K intake among Italians due to an inadequate consumption of fruits, vegetable foods and legumes, which were once the major protein source of the traditional Mediterranean diet.^39^ Two tablespoons of PCAs of 30 g have 150 mg K, and therefore the PCA obtained could be considered a good source of K.^40^

### Texture characteristics of PCA‐LG, PCA‐MG and PCA‐HG

The preparation of cream or spreadable cheese consists of blending natural cheese with other ingredients that could act as calcium‐chelating agents promoting disaggregation of the protein in the cheese matrix, and where the fat is contained as an emulsion within the matrix.^41^, ^42^ During oral processing, cream cheese does not fracture, but some structural breakdown occurs as a result of work between the tongue and hard palate, mouth temperature and product contact with saliva.^43^ A nonoral textural characteristic, the spreadable index, is a key cream cheese attribute for consumers.^44^ In our study, the higher concentration of EFG resulted in a product with higher spreadability index and lower hardness, compared to the PCA produced with the lowest concentration of EFG. This was probably related to the higher presence of fat in the formulation, which led to a softer structure. The textural behavior of 150 g kg^−1^ EFG was able to confer to the final product a spreadable index similar to that of DC, without the need to add a high saturated fat. However, our PCAs were more elastic and less hard than DC. Our results agreed with a previous study on the addition of peas in different concentration to milk yogurt, in which the resulting products had lower hardness compared to a reference dairy yogurt.^45^ On the other hand, our results disagreed with the findings of Moon *et al*.,^46^ who successfully replaced the palm oil usually used in commercial NDC with an oleogel made with canola oil and carnauba wax in the preparation of a hard NDC using soy protein isolates. The discrepancy in the results is probably due to the different gel composition used.

In DC, fat is the main component affecting the rheological and sensory characteristics of commercial spreadable cheeses. The low‐fat creamy cheese versions are usually perceived to be tougher and less spreadable than their full‐fat equivalents.^47^ The low‐fat content and high protein content of our products might have negatively affected the breakdown of the cream in the mouth. In fact, this was translated by the high values of chewiness and gumminess of the formulated PCAs, obtained by the TPA, compared with DC, and in the mean score ‘low’ assigned by the panelists to the attribute ‘solubility’ in the sensory evaluation. However, panelists also assigned a ‘low’ score to the attributes ‘sticky’ (0.85–1.38) and ‘gumminess’ (0.62–1.23). This discrepancy in the results between the TPA and the sensory analysis highlights the importance of conducting a sensory evaluation when developing a new food product.

### Volatile profile and sensory features of PCAs

Different pathways could lead to the development of volatile compounds, e.g. microbial activity, heating treatments, fat oxidation, proteolysis and other enzymic activities. For this reason, a first comparison between the VOC profile of the slurry and the plain PCA‐HG, without spices, was done with the aim of understanding the impacts of the addition of EFG to the slurry and the additional treatment at 40 °C for 20 min on the volatile profile of PCA‐HG.

Many studies on pulse crops showed a different evolution of the aroma when heat treatment was applied, mainly due to the inactivation of the lipoxygenase enzyme, which is one of the factors responsible for development of off‐flavors.^48^, ^49^ Fahmi *et al*.^50^ reported a significant decrease in the concentration of some compounds, such as pentanal, 1‐penten‐3‐ol, hexanal and heptanal, in response to the thermal treatment of yellow pea. The slurry was characterized by a lower total amount of volatile compounds developed consequent to the lipoxygenase activity in respect to PCA‐HG, probably due to the slurry preparation process, which involves thermal treatment at 80 °C for 40 min, which could have inactivated the lipoxygenase enzyme and helped the volatilization of the already present VOC.

The heating temperature (40 °C) of PCA‐HG is within the range of the optimal conditions for the activity of lipoxygenase.^51^ This could have led to the development of the oxidized compounds. Simultaneously, EFG addition enriched the matrix with the unsaturated fatty acids of EVOO (e.g., oleic and linoleic acids), which could be oxidized chemically or enzymatically by lipoxygenase activity, producing by‐products such as octanal, heptanal (oleic and linoleic acid oxidation), pentanal, nonanal, 2‐heptenal, hexanal and 1‐hexanol (from linoleic acid) or 1‐octen‐3‐ol and 2‐hexenal (from either linoleic or linolenic acid). Most of these molecules were significantly more abundant in PCA‐HG than in the slurry. Such a phenomenon could lead to a possible off‐flavor formation – the so‐called beany flavor – and for this reason the addition of spices was considered to cover the unpleasant odors.^9^, ^52^

As expected, the addition of these spices led to a richer volatile profile for both PCA‐HGO and PCA‐HGR. Many authors focused on their addition as flavoring ingredient, as well as their antioxidant activity.^53^, ^54^ In fact, most of the VOC of the formulated products were in accordance with those found in previous studies and are the typical molecules of oregano and rosemary spices.^55^ Thymol methyl ether, carvone, carvenone, terpinen‐4‐ol, α‐ylangene, *p*‐cymen‐7‐ol and 1‐heptanol were found only in PCA‐HGO samples,^55^, ^56^ while *trans*‐carveol, 1‐penten‐3‐ol ethyl vinyl carbinol, α‐terpineol, pinocarvone, d‐limonene, 3‐carene and α‐pinene were found only in PCA‐HGR.^55^, ^57^ All these typical VOC could be the compounds giving, floral, spicy, earthy, green and herbal notes.^58^, ^59^ However, we also found many other VOC which could have contributed to the final aroma of the product. Among these, PCA‐HGR had the highest abundance of 2,3‐dimethylpentanal, hexanal, 2‐pentenal, 2‐heptenal, octanal, 1‐octen‐3‐ol, 2‐ethyl‐1‐hexanol, benzyl alcohol, ethyl acetate, 6 methyl‐5‐hepten‐2‐one and acetic acid, which could confer green, fatty, fruity, pungent and earthy notes, and PCA‐HGO had the highest abundance of 2‐octenal, 2‐nonenal, 3‐hexen‐1‐ol, 1‐heptanol and α‐phellandrene, which could confer fatty, green, herbal, terpenic and woody odor notes.^60^, ^61^ Finally, PCA‐HG had the highest abundance of decanal, ethanol, 1‐penten‐3‐ol, maltol, dimethyl disulfide and γ‐caprolactone, giving theoretically pleasant odors of floral, fatty, citrus, alcoholic and grass, and the unpleasant odors of rancid and sulfur.^62^

The sensory evaluation results confirmed that the unpleasant flavor (legumes or beany flavor) was masked by the addition of spices as the mean score of spices was ‘mild’, while the mean scores of the legumes was ‘low’ and sourness and astringent were absent in the samples. The addition of oregano conferred a touch of bitterness to the final product. All samples were perceived more sweet than salty, even though the sweetness score was ‘mild’. The addition of both spices also conferred a lumpy texture to the final product, which could be resolved by reducing the particle size (while grinding) of the added spices. All our samples had an equilibrated consistency as all textural attributes scored ‘low’.

## CONCLUSIONS

The use of dry‐fractioned pea protein concentrates combined with EFG with inulin and EVOO allowed us to develop, on a laboratory scale, a clean label and nutritious spreadable plant‐based cheese analogue. The formulated products had a higher content of protein (134 g kg^−1^) and a lower level of fat (52 g kg^−1^) than the plant‐based cheese analogues available on the market.

The use of 150 g kg^−1^ EFG conferred to the final product developed a spreadability index similar to that of commercial dairy cream cheese. The addition of spices allowed masking of the typical beany flavor given by the dry‐fractionated pea without any adverse sensorial characteristics. However, only when oregano was added to the product was a bitter taste recorded by the panelists. Expanding the plant‐based products selection with alternatives characterized by a clean label and a higher nutritional value will better accommodate a broader audience of ‘healthy’ consumers aware of the importance of the plant‐based diet. Finally, the ingredients and product concepts developed in this study could be used in the food industry to upgrade the formulation of plant‐based cheese on a larger scale.

## AUTHOR CONTRIBUTIONS

Conceptualization and design: CS, MM and MF; data acquisition, analysis and interpretation: MM, DD and GN; drafting the manuscript: MM, DD and GN; writing – review and editing: CS, MM, AP, FC and MF; finalizing: CS, MM and MF; supervision: CS and MF.

## CONFLICT OF INTEREST

The authors declare no conflict of interest.
